# Sex Differences in Cardiovascular Disease and Cognitive Impairment: Another Health Disparity for Women?

**DOI:** 10.1161/JAHA.119.013154

**Published:** 2019-09-24

**Authors:** Annabelle Santos Volgman, C. Noel Bairey Merz, Neelum T. Aggarwal, Vera Bittner, T. Jared Bunch, Philip B. Gorelick, Pauline Maki, Hena N. Patel, Athena Poppas, Jeremy Ruskin, Andrea M. Russo, Shari R. Waldstein, Nanette K. Wenger, Kristine Yaffe, Carl J. Pepine

**Affiliations:** ^1^ Section of Cardiology Department of Medicine Rush Medical College Chicago IL; ^2^ Barbra Streisand Women's Heart Center Smidt Heart Institute Cedars‐Sinai Medical Center Los Angeles CA; ^3^ Departments of Neurological Sciences Rush Alzheimer's Disease Center Rush Medical College Chicago IL; ^4^ Division of Cardiovascular Disease Department of Medicine University of Alabama at Birmingham AL; ^5^ Section of Cardiology Department of Medicine Stanford University Palo Alto California; ^6^ Department of Cardiology Intermountain Heart Institute Intermountain Medical Center Salt Lake City UT; ^7^ Department of Translational Neuroscience Michigan State University College of Human Medicine Grand Rapids MI; ^8^ Department of Psychiatry, Psychology and Obstetrics & Gynecology University of Illinois at Chicago IL; ^9^ Section of Cardiology Department of Medicine Brown University School of Medicine Providence RI; ^10^ Division of Cardiology Massachusetts General Hospital Boston MA; ^11^ Section of Cardiology Department of Medicine Cooper Medical School of Rowan University Camden NJ; ^12^ Department of Psychology University of Maryland, Baltimore County Baltimore MD; ^13^ Section of Cardiology Department of Medicine Emory University School of Medicine Atlanta GA; ^14^ Department of Psychiatry, Neurology and Epidemiology University of California San Francisco San Francisco CA; ^15^ Division of Cardiovascular Medicine Department of Medicine University of Florida Gainesville FL

**Keywords:** Alzheimer disease, APOE, cardiac procedures, complication, dementia, ischemic heart disease, strokes, Aging, Cardiovascular Disease, Risk Factors, Women, Genetic, Association Studies

## Introduction

Although the number of Americans dying of cardiovascular disease (CVD) continues to increase since 2010 after decades of decrease, advances in the management of CVD have led to increased longevity among both women and men, with more people, mostly women, now surviving into their 80s and beyond.^1^ Paralleling this increased longevity, however, is an increasing prevalence of, and mortality from, neurodegenerative cognitive disorders.^1^ These cognitive disorders include dementia, a syndrome that has a multitude of causes and symptoms that ultimately have substantial impact on social and occupational activities and aspects of daily living.^2^ Typical symptoms of dementia include changes in memory, problem solving, language, and executive functioning.^2^ Although there are often distinct patterns, symptoms, and specific brain pathology associated with different dementias, multiple autopsy studies are now demonstrating that people with symptoms of a dementia will often have multiple brain pathologies noted at autopsy that were associated with the dementia.^3^, ^4^ Multiple reports document that approximately two thirds of those clinically diagnosed with Alzheimer dementia are women.^5^ Furthermore, it is estimated that by 2040 the number of Americans with some form of cognitive impairment (CI), including dementia, will be ≈8.3 million women and ≈3.3 million men.^5^ This sex‐related CI disparity is concerning and raises important questions about its possible relation to CVD and CVD‐related risk factor conditions.

Although there are multiple types of dementia syndromes with different cognitive, behavioral, and pathological characteristics, the most common types are Alzheimer dementia and vascular cognitive impairment.^6^ Alzheimer dementia is characterized by an irreversible, progressive disorder that slowly destroys memory and thinking skills, and eventually the ability to perform simple tasks.^7^ Vascular cognitive impairment, a term that encompasses all levels of CI from its mildest form to vascular dementia, is characterized by progressive changes in behavior, function, and cognition caused by vascular injury (eg, strokes [infarcts], microbleeds [cerebral amyloid angiopathy and microhemmorhages]) or disease within the brain and includes deficits in executive functioning, attention, and language. This is in contrast to episodic memory deficits, which are more prominent in Alzheimer dementia. Increasingly, the term “mixed dementia” is used to describe the coexistence of both Alzheimer disease (AD) and vascular neuropathology in people experiencing dementia symptoms.^8^

In 2018, new criteria for AD were established by the National Institute on Aging and Alzheimer's Association to recognize that the disease occurs across a continuum of pathologic changes that precede its clinical manifestations and culminates with Alzheimer dementia.^9^ The clinical syndrome is noted as Alzheimer dementia, and the term dementia caused by AD is used when neuroimaging biomarkers or biofluid markers or autopsy confirm the diagnosis. This distinction continues to highlight the ongoing research that shows that AD begins many years before the symptoms of Alzheimer dementia are present.^10^ The term mild cognitive impairment (MCI) is used in people with demonstrable CI who have not crossed the threshold to dementia. MCI can refer to either a predominantly memory (amnestic) or nonmemory (nonamnestic) cognitive deficit and can be the initial symptom of a dementia syndrome, but it may also be secondary to other conditions or disease processes.^11^ A meta‐analysis revealed that women have a higher prevalence of nonamnestic MCI but suggested no sex‐related differences in the incidence or prevalence of amnestic MCI.^12^

At age 45 years, the lifetime risk for developing Alzheimer dementia is estimated at 1 in 5 for women versus only 1 in 10 for men.^13^ Possible reasons for this sex disparity, alone or more likely in combination, include greater longevity of women; their higher incidence of chronic diseases (particularly those CVD linked with CI), genetic predispositions, differences in cognitive testing performance (women outperform men on tests of verbal ability)^14^ (thereby potentially “masking” any underlying cognitive deficits, resulting in a later presentation to the healthcare provider for complaints of change in cognitive function), socioeconomic, psychosocial, and biological factors.^15^ Education level and occupational attainment (eg employment) can impact cognitive resilience and explain some of the sex disparities noted in dementia.^16^, ^17^ Traditionally, older women may not have had as many opportunities for attaining a higher level of education nor an equal period of time in the workforce compared with men, thus possibly putting them at an increased risk for developing cognitive decline and dementia. However, results from studies continue to be mixed, as methodological issues, study design, and comparability across studies remain difficult. Lastly, among the psychosocial factors, caregiving has emerged as a potential risk factor for developing dementia. Sex differences in spousal care are highly prevalent for patients with dementia, with women delivering the majority of the care and spending more time devoted to care.^18^ The increased stress of caregiving, coupled with lower levels of social support and poor physical health, are increasingly being examined as strong risk factors for cognitive decline and dementia in female caregivers.

In addition to the aforementioned contributing factors of sex‐related differences in the dementia syndromes, this review examines sex differences in CVD and a broad range of CVD risk factors that may contribute to CI to explain, at least in part, the higher prevalence of dementias in women versus men. Potential implications of these sex differences are addressed in context for practitioners, our healthcare system, and related resource consumption. Important knowledge gaps are identified to direct the focus of future research.

The American College of Cardiology CVD in Women Committee identified this topic and invited experts in the fields of CVD and/or CI to contribute sections in their respective fields. They did their own literature searches and submitted their sections to the chair of the writing committee (ASV), who compiled and summarized the sections to create a cohesive document. Over 300 selected publications were reviewed, and those addressing sex differences or new information were included in this document.

This document is a summary of the topics that include stroke, microvascular or small‐vessel disease, and sex‐specific issues about differences in CVD that may help explain the increased prevalence of CI and dementia in women.

## Causes of Cerebrovascular Accident/Brain Ischemia, and Infarct

### Hypertension

A substantial literature documents that higher blood pressure (BP), a key risk factor for stroke, as well as myocardial infarction and heart failure (HF), also presage vascular dementia, AD, and earlier forms of vascular CI.^19^, ^20^, ^21^, ^22^ In addition, higher BP is associated with lower levels of cognitive function before evidence of frank “impairment.”^23^ This process occurs over the life span,^20^, ^24^ starting in childhood,^25^ and exhibits a long and insidious course. Higher childhood systolic BP has been associated with lower levels of cognitive function in midlife,^26^ and higher midlife BP predicts less favorable later‐life cognitive outcomes.^19^, ^20^ However, among the elderly, a reversal of association shows that higher BP often relates to better cognitive outcome.^20^ Men have a higher prevalence of hypertension versus women until about age 64 years; however, after that age the prevalence of hypertension in women is higher than in men.^1^ Data generally suggest that both high and low BP are associated with reduced cognitive function and greater dementia risk.^19^, ^20^

Impairment in multiple cognitive domains is associated with higher BP, although the preponderance of evidence suggests that executive functions, perceptual motor speed, and learning and memory may be most affected.^20^ However, patterns of affected cognitive domains (including null findings) vary greatly by study. This is not surprising given pronounced methodological differences among studies, variations in BP measurement and risk factors, heterogeneity among hypertension patients, and as discussed later, the likelihood of multiple underlying brain mechanisms with potentially differing impacts on cognitive function. This striking admixture of results may also reflect the influence of vulnerability and resilience factors.^24^, ^27^ In that regard, studies of effect modification suggest more pronounced BP‐related risk is conferred at younger ages, lower levels of education, presence of APOE ε4 alleles, and in conjunction with other CVD risk factors.^19^, ^20^, ^24^, ^27^ Those receiving antihypertensive medication appear to fare better longitudinally.^28^ A review of randomized trials of antihypertensive agents for prevention of either cognitive decline or dementia suggests that risk reduction can be achieved with some select drug classes.^28^

Proposed mechanistic pathways linking elevated BP to cognitive decline and dementia are numerous and include structural and functional brain mechanisms that promote both ischemic injury and expression of AD neuropathology.^19^, ^20^, ^21^, ^22^ With standard neuroimaging procedures, higher BP has been associated with decreased global and regional cerebral blood flow and metabolism, brain atrophy, white matter lesions, subclinical brain infarction, lesser white matter microstructural integrity, and altered functional connectivity and activation patterns. Studies in predominantly animal models suggest mechanistic pathways that include structural changes to cerebral vessels, atherosclerosis, vascular remodeling and stiffening, small‐vessel disease, microvascular rarefaction, endothelial dysfunction, diminished neurovascular coupling and autoregulatory function, and alterations in the blood–brain barrier. Higher BP has also been associated with increased expression of AD neuropathology, which includes brain β‐amyloid levels, neuritic plaques, and neurofibrillary tangles.^19^, ^20^, ^21^, ^22^

The SPRINT (Memory and Cognition in Decreased Hypertension) MIND study is the first randomized trial to investigate the cognitive effects of intensive BP lowering (<120 mm Hg systolic BP goal) versus a traditional goal of <140/90 mm Hg. In adults ≥50 years old without diabetes mellitus or prior stroke, intensive BP lowering significantly reduced the rate of MCI (14.6 versus 18.3 cases per 1000 person‐years; hazard ratio [HR], 0.81; 95% CI, 0.69–0.95) and the combined rate of MCI or probable dementia (20.2 versus 24.1 cases per 1000 person‐years; HR, 0.85; 95% CI, 0.74–0.97).^29^ Furthermore, a preliminary report of their brain magnetic resonance imaging (MRI) substudy documented significantly reduced development of white matter lesions,^29^, ^30^ supporting the hypothesis of a microvascular mechanism. These are remarkable findings considering that the population enrolled in SPRINT was at low risk for CI, as patients with diabetes mellitus or prior stroke were excluded, and follow‐up was terminated early (only 3.26 years, median) because of significant 27% reduction in all‐cause mortality with the lower BP goal. However, the Alzheimer's Association awarded support for SPRINT MIND 2.0 that will extend follow‐up for 2 additional years to determine effects on “probable dementia” alone. Whether significant sex differences in development of MCI, dementia, or white matter hyperintensity lesions will emerge in subsequent analyses would be very important.

These studies reveal biologically plausible pathways to vascular dementia, Alzheimer dementia, and mixed forms of dementia among those with higher BP and may help explain varying patterns of cognitive correlates noted across investigations. Although several investigations suggest sex differences, findings are mixed regarding the relative vulnerability of women versus men.^31^, ^32^ Variation in the course of CVD among women and men suggests that interactions of sex and age are important considerations relative to CI. An interaction between hypertension and menopausal status indicated that cognitive performance was worse in hypertensive, versus normotensive, postmenopausal women. Notably, this finding was not present in premenopausal women,^33^ suggesting the importance of examining hormonal and other influences that may be operative within different samples of women.

### Atherosclerosis

#### Intracranial large artery disease

Severe atherosclerosis is recognized as a potent risk factor for CI.^34^ Large‐artery intracranial occlusive disease remains the most common stroke subtype.^35^ This subtype may occur more frequently in women than men^36^ and progresses rapidly after menopause.^37^ Among women, hypertension and diabetes mellitus are predictors of intracranial internal carotid artery calcification or intracranial atherosclerosis,^38^ and women with severe large‐artery intracranial occlusive disease, defined as ≥70% stenosis, appear to have a higher risk of recurrent stroke than men.^39^ To our knowledge, no large‐scale studies have examined sex differences in cognitive function among people with large‐artery intracranial occlusive disease.

#### Cerebrovascular small‐vessel disease

While hypertension, hyperlipidemia, and diabetes mellitus have long been recognized as risk factors for stroke and vascular dementia, epidemiological data suggest that they are also associated with increased incidence of cognitive decline^40^ and clinically diagnosed Alzheimer dementia.^41^ Vascular disease risk factors are thought to accelerate the development of AD neuropathology (eg, accumulation of β‐amyloid and neurofibrillary degeneration). Alternatively, CVD‐related brain injury may interact additively (or synergistically) with AD neuropathology to cause earlier expression of dementia.

Neuropathology studies note that most people with dementia who die have substantial cerebrovascular disease (small‐vessel disease, cerebral microbleeds, and microinfarcts)^42^, ^43^ and axonal damage.^44^ MRI imaging documents damage from small‐vessel disease such as white matter lesions and lacunar infarcts. Atherosclerotic calcification is strongly associated with these MRI‐markers of subclinical vascular brain disease.^45^ Although no studies report on sex differences in intracranial small‐vessel disease, it well known that women are more likely than men to have nonobstructive multivessel disease and more microvascular dysfunction.^46^ There is considerable overlap between risk factors for vascular disease and for cognitive decline.^40^, ^42^, ^43^ The shared risk factors for CVD and the clinical development of Alzheimer dementia are associated with reduced cerebral glucose uptake and reductions in blood flow, which together are thought to be associated with increased accumulation of cerebral β‐amyloid over time.

Recent advances in MRI have shown that “breakdown” of the blood–brain barrier is a core mechanism in cerebral small‐vessel disease and dementia.^47^ It is unclear whether the biological mechanisms involved in disrupting the blood–brain barrier are similar to those associated with coronary or other organ microvascular dysfunction. Establishing whether these relationships exist may be an important research direction to better understand links between CVD and CI/dementia.

#### Coronary atherosclerosis–nonobstructive (coronary artery disease)/coronary microvascular disease

Coronary artery calcification scores have been associated with dementia and cognitive decline. A population‐based study of 5764 men and women aged 66 to 86 years born between 1907 and 1935 found that an increasing coronary artery calcification score was associated with higher rates of dementia and lower executive function scores. Brain volumes (ie, gray matter, white matter, and total brain tissue volumes by MRI) were decreased with increasing coronary artery calcification.^48^

On the contrary, the Rotterdam Study followed 7983 people to evaluate determinants of disease in those ≥55 years old.^49^ Nonenhanced computed tomography chest imaging, including proximal vessels of the head, was performed in 1847 subjects. Non–coronary artery calcifications, but not coronary artery calcification, were significantly associated with increased rates of dementia: extracranial carotid arteries (HR 1.39; 95% CI 1.09; 1.77); aortic arch (HR 1.38; 95% CI 1.02; 1.86); and intracranial carotid arteries (HR 1.31; 95% CI 1.01; 1.70). The authors postulated that more coronary artery calcification caused increased CVD events, including death, so those subjects may not have survived long enough to develop dementia.

Women more frequently have nonobstructive coronary artery disease (CAD), coronary spasm, and microvascular or small‐vessel disease than men.^46^, ^50^ Both nonobstructive CAD and microvascular disease may contribute to increased risk of CI and dementias in women.^51^ Most diseases caused by microvascular dysfunction/disease have no evidence‐based treatments documented to be effective, but potential treatments are being investigated.

An association between CAD and CI has been observed in men, but not in women^52^; however, this sex difference was no longer statistically significant with increasing age (n=1969; aged 70–89 years).^53^ Although no sex interaction was reported, after acute myocardial infarction, CI was associated with less invasive care, less referral and participation in cardiac rehabilitation, and worse risk‐adjusted 1‐year survival.^54^

### Atrial Fibrillation

In an observational analysis of 35 608 patients without either atrial fibrillation (AF) or dementia, the 5‐year rate of AF in men was 14.0% versus 11.9% in women (*P*<0.0001).^55^ Despite higher rates of AF in men, it appeared that women may have slightly higher 5‐year rates of dementia (1.1% versus 0.9%, *P*=0.09).^55^ Among patients who developed AF, women appeared to have a slightly higher incidence of dementia compared with men (3.7% versus 3.0%, *P*=0.11). In this analysis, older age, diabetes mellitus, prior stroke, and kidney disease predicted dementia in women without AF, and greater age, prior stroke, and CAD predicted dementia in women with AF.^55^ In meta‐analyses of AF patients with stroke, risk ratios for CI or dementia range from 2.43^56^ to 2.70.^57^ After a stroke, women have worse long‐term functional outcomes with reduced quality of life, compared with men.^58^ Although sex‐specific data regarding long‐term risk of CI after a stroke are lacking, a stroke registry showed that women had significantly lower cognitive function scores than men on a stroke‐specific quality‐of‐life scale; (2.8 versus 3.4, *P*<0.001).^59^

**Figure 1:**
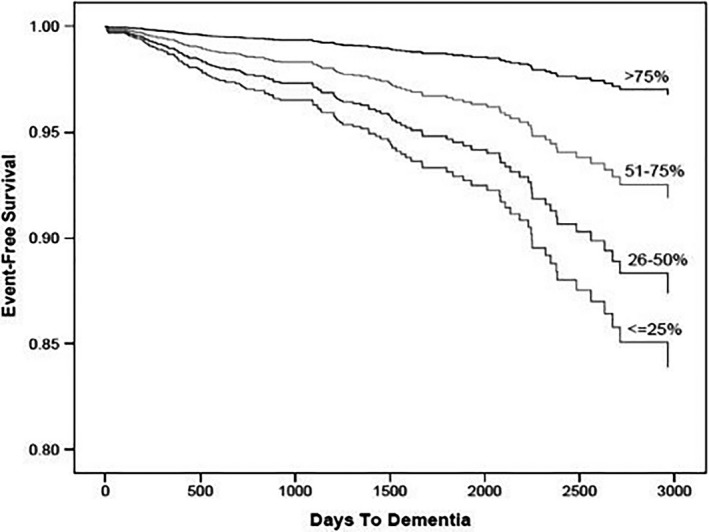
Event‐free survival estimates for dementia incidence among categories of percent time anticoagulation with vitamin K antagonists is in the therapeutic range. Reprinted from Jacobs et al^60^ with permission. Copyright ©2014, Elsevier.

Observational studies in AF patients indicate that “time in therapeutic range” of anticoagulation is inversely associated with dementia risk (Figure 1).^60^ Interestingly, increased dementia risk was observed with both under‐ and overanticoagulation.^60^ Although direct oral anticoagulants have not been studied in randomized controlled trials versus warfarin to study the effect of anticoagulation on CI and dementia, observational data (41% women) suggest a lower long‐term risk of stroke and dementia with these agents.^61^ Underuse of anticoagulation remains a problem in AF and is more frequent in women versus men,^62^ despite observational data indicating that warfarin lowers stroke risk more in women.^63^ Improving prompt anticoagulant use in women with AF is critical, as delays in initiation incrementally increase dementia risk.^64^ Although sex differences were not evaluated, AF was associated with a >2‐fold increased risk of developing silent cerebral infarction, detected by MRIs.^65^ These subclinical cerebral ischemic events are associated with CI despite use of oral anticoagulants in up to 88% of AF patients.^66^

In a retrospective registry study of almost half a million AF patients, those treated with anticoagulants at baseline had a 29% lower risk of dementia versus patients without anticoagulant treatment (HR 0.71, 95% CI 0.68–0.74). When analyzed “on anticoagulant treatment,” there was a 48% lower risk (HR 0.52, 95% CI 0.50–055), and no sex‐related differences or differences between new oral anticoagulants and warfarin were observed.^67^

Sleep apnea and sleep‐disordered breathing can increase the risk of AF.^68^ Whether sleep apnea causes CI has been investigated; a pooled analysis of 7 cross‐sectional studies suggested that those with sleep‐disordered breathing had slightly worse executive function (standard mean difference, −0.05; 95% CI −0.09 to 0.00) but was not associated with global cognition or memory.^69^ No sex differences were analyzed in this report. However, a nationwide survey confirmed sex differences in the association between various self‐reported sleep disturbances and CI in people aged 65 years and older. They found that there was a significant interaction between sex and difficulty breathing during sleep on CI. In men, difficulty breathing during sleep, habitual snoring, and prolonged sleep duration (>8.5 hours) correlated significantly with CI. In women, only prolonged sleep duration (>8.5 hours) was associated with higher likelihood of CI.^70^

### Heart Failure

About 30% to 80% of HF patients experience some degree of CI, depending on study design, HF severity, patient age, sample size, and diagnostic criteria.^71^ Most HF patients have MCI, a quarter may have moderate‐to‐severe CI,^72^ and severity may fluctuate over time, either improving with effective HF treatment or progressing to dementia.^71^ The role of sex in CI development among HF patients, however, is unclear. In a cross‐sectional analysis of Medicare claims data for individuals ≥67 years (58% women), sex did not predict moderate‐to‐severe CI.^73^

HF with preserved ejection fraction (HFpEF) was the most prevalent form of HF in older women, and these patients had higher risk of subclinical cerebral infarction, even without AF, than those without HFpEF.^74^ In patients with HFpEF but no AF, only male sex (odds ratio 2.26, 95% CI 1.12–4.58) and a larger left atrium (odds ratio 1.41, 95% CI 1.00–2.00) were associated with increased odds of having subclinical cerebral infarction after multivariable adjustment. Those with HFpEF and subclinical cerebral infarction had lower cognitive scores, noted in tests for executive functioning and episodic memory, than the reference (no HFpEF/no subclinical cerebral infarction) and HFpEF/no subclinical cerebral infarction groups.^75^ Cognitive performance by sex was not reported.

Multiple studies in HF patients document a strong, independent association between CI and increased mortality and hospital readmissions. Intact executive function and memory are needed to identify worsening symptoms, adhere to self‐care practices such as medication regimens and lifestyle modifications, maintain clinic visits, and comply with dietary recommendations. No clear association has been identified between sex and medication adherence among HF patients with CI.^76^

### Cardiovascular Interventional Procedures

Sex differences in cardiac interventions such as catheter‐based procedures and surgeries were evaluated for sources of cerebral microinfarcts and strokes that can contribute to CI.

#### Cardiac procedures

Cardioembolic strokes are a well‐recognized complication of many invasive cardiovascular procedures. Invasive cerebral and coronary angiography, coronary artery bypass graft surgery (CABG), surgical aortic valve replacement, and transcatheter aortic valve replacement (TAVR) may cause both acute and, even more frequently, subacute, “subclinical” microinfarcts (Table 1).^77^ For TAVR, women generally have higher Society of Thoracic Surgeons risk scores, more bleeding, and more vascular complications but similar stroke rates with improved 1‐year, all‐cause mortality.^78^ With surgical aortic valve replacement, women appear to have similar stroke rates, but higher in‐hospital mortality.^79^

**Table 1:** Estimated Annual US Patients With New Brain Lesions After Vascular Procedures

Procedures	No. of Annual US Patients	Incidence of New Brain Lesions, %	No. of Annual Patients With New Brain Lesions
Coronary angiography	1 072 000	11–17	118 000–182 000
Percutaneous coronary intervention	596 000	11–17	66 000–101 000
CABG	242 000	16–51	39 000–123 000
Surgical aortic valve replacement	90 000	38–47	34 000–42 000
AF ablation	72 000	8–18	6000–13 000
Transcatheter aortic valve implantation	10 000	68–91	7000–9000
Carotid endarterectomy	93 000	4–34	4000–32 000
Carotid artery stenting	70 000	15–67	11 000–47 000
Cerebral angiography	300 000	11–20	33 000–60 000
Endovascular aneurysm	30 000	10–64	3000–19 000
Total	2 600 000	13–24	321 000–628 000

AF indicates atrial fibrillation; CABG, coronary artery bypass graft.

Reprinted from Gress^77^ with permission. Copyright ©2012, Elsevier.

With TAVR, the increase in overt (4%) and silent (70%)^80^ stroke risk may be offset by the increase in forward blood flow, with significant and sustained improved executive functioning in most patients.^81^ However, female sex was a predictor of increased clinical neurologic events after TAVR.^81^ A review of TAVR in women concluded that, although women had better long‐term survival versus men, women had increased stroke risk.^82^

#### Implantable cardioverter defibrillator implantation procedures

There are several potential central nervous system risks associated with defibrillation testing during implantation of implantable cardioverter defibrillators (ICDs). These include risks related to ventricular fibrillation itself, which may lead to circulatory arrest and hypoperfusion, and thromboembolic events resulting from shock therapy. In addition, adverse effects related to anesthetic drugs required during implantation or testing undoubtedly contribute to adverse neurological effects. However, data on sex‐specific differences of these risks are lacking.

After repeated ventricular fibrillation episodes, cumulative quantitative electroencephalographic depression (a sign of cerebral ischemia) was associated with ICD testing.^83^ However, others found a negative correlation between the number of tests and recovery time.^84^ The mean reperfusion time interval between episodes of ventricular fibrillation induction was shorter in patients with CI versus those without, and there was correlation between circulatory arrest time and electroencephalographic recovery time.^84^ However, the small number of patients in these studies (9–37) precludes evaluation of sex differences and definitive conclusions. Nevertheless, it is now standard to wait at least 5 minutes between ventricular fibrillation inductions.

Cognitive assessments were done in patients receiving ICDs for primary prevention to evaluate CI after appropriate ICD events for ventricular fibrillation. Over 26‐month follow‐up, 15 appropriate shocks were observed in 7 patients, and among those with appropriate shocks, defibrillation was an independent predictor of poor cognitive function, along with age and education.^85^ However, this association does not prove a “cause–effect” relationship as factors other than those examined may contribute. Psychocognitive improvement has been noted following ICD implantation with cardiac resynchronization devices, presumably related to improved cardiac function.^86^

Thromboembolic complications may also be associated with defibrillation testing. Shocks, with restoration of sinus rhythm in the presence of intracardiac thrombi, may result in thromboembolic complications such as stroke, microinfarcts, and associated CI. If defibrillation testing at the time of ICD implantation is planned for persistent AF patients not adequately anticoagulated, preoperative transesophageal echocardiography is indicated to exclude pre‐existing thrombus. To avoid cerebral hypoperfusion, induction of ventricular fibrillation for ICD testing is no longer done with contemporary ICD technologies.

#### Surgical procedures requiring cardiopulmonary bypass

Earlier studies showed that sex is an independent predictor of operative mortality after CABG,^87^ but women are often referred for surgery later in the course of their disease, or for more urgent CABG, which may partially explain the higher mortality risk.^88^ More recent studies such as the STICH Trial (Surgical Treatment for Ischemic Heart Failure) showed no sex differences in mortality.^89^ However, women undergoing CABG or valve surgery were more likely than men to have postoperative neurological events after 30 days (HR 1.21, 95% CI 1.14, 1.28) that were not explained by known risk factors.^90^

While stroke is reported in 2% to 4% of patients following cardiac surgery, postoperative CI is much more frequent, with a variable incidence depending on definition and time after surgery.^90^ After CABG, CI incidence was 53% at discharge, 36% at 6 weeks, 24% at 6 months, and 42% at 5 years; importantly, CI at discharge predicted long‐term CI in both women and men.^91^

A meta‐analysis of 13 randomized trials (2405 patients) found no significant differences between on‐ and off‐pump surgery in postoperative psychometric tests, suggesting that cardiopulmonary bypass may not be a major cause of postoperative CI.^92^ Advanced age, longer aortic cross‐clamp duration, and pre‐existing CI are risk factors for postcardiac surgery CI.^93^ After CABG, overall CI frequency was not different between women and men in a study using several cognitive domains and neuropsychological tools; however, women had poorer performance postoperatively on visual‐spatial tasks.^94^

### Sex‐Specific Risks

#### Preeclampsia

A meta‐analysis of 13 studies (1314 women with prior preeclampsia and 289 080 women with prior normotensive pregnancy) evaluated the relationship of preeclampsia and CI; however, the median time since pregnancy was only 6 years. Pooling of cognitive outcome measures for studies assessing brain imaging or a clinical diagnosis of dementia were limited by differences in reporting and marked heterogeneity between studies. It was concluded that although preeclampsia is associated with subjective cognitive symptoms, the review did not clearly demonstrate an association with preeclampsia and CI on standard neurocognitive tests.^95^

#### Hormone replacement therapy and dementia: role of cardiovascular risk factors

The WHIMS (Women Health Initiative Memory Study) of women ≥65 years old is the only randomized, placebo‐controlled trial of menopausal hormone therapy for primary prevention of dementia.^96^ Women with a uterus (n=2947) were randomized to receive oral conjugated equine estrogen (CEE; 0.625 mg/d) plus medroxyprogesterone acetate (MPA; 2.5 mg/d CEE/MPA) or placebo. Women without a uterus (n=4532) were randomized to receive oral CEE (0.625 mg/day) or placebo. Compared with placebo, CEE/MPA doubled the risk for all‐cause dementia, but CEE alone had no effect and these outcomes were not modified by smoking, CVD, stroke, diabetes mellitus, hypertension, or statin or aspirin use.^96^ Similar to dementia findings, CVD risk factors did not modify effects of hormone therapy on global cognitive function.

In a subgroup of WHIMS (n=1424) who underwent brain MRI, ischemic brain volume (a measure of subclinical cerebrovascular disease) did not differ between hormone therapy and placebo groups, suggesting that ischemic changes were not the main mechanism underlying the observed increase in all‐cause dementia risk.^97^ However, consistent with the stroke risk associated with hormone therapy found in the Women's Health Initiative,^98^ rates of accumulation in white matter lesion volume and total brain lesion volume were higher among women with a history of CVD treated with hormone therapy versus placebo.^97^

In contrast to the WHIMS, an 18‐year observational follow‐up study of 27 347 women in the Women's Health Initiative found a lower risk of mortality from Alzheimer dementia or dementia with CEE versus placebo, but no effect of CEE/MPA.^99^ Mortality findings should be interpreted with caution due to the nonrandomized study design, and because the cause of death was determined by National Death Index search, or in some cases contacting next of kin, whereas 48% to 55% of dementia cases were prospectively adjudicated in WHIMS. Given that hormone therapy reduces diabetes mellitus risk, and diabetes mellitus is associated with increased risk of Alzheimer dementia and dementia,^100^ additional investigation is needed to determine whether hormone therapy‐associated reductions in mortality from dementia are related to menopausal hormone therapy–associated reductions in diabetes mellitus.

Overall, there is limited evidence from the Women's Health Initiative to support the view that CVD risk factors or CVD history mediate effects of hormone therapy on dementia or cognition.

### Cardiac Medications and Anti‐Inflammatory Drugs

Many medications can affect cognition, either directly by altering neural processes or indirectly by reducing perfusion pressure or inducing hypoglycemia or hyponatremia. Older individuals are particularly susceptible, in part because of polypharmacy and age‐related alterations in drug metabolism. Older women may be more susceptible than men because of smaller stature, because less muscle mass may mask the severity of renal impairment.

A detailed review of all cardiovascular medications is beyond the scope of this review, but available data show that aspirin, statins and other lipid‐lowering agents, β‐blockers, modulators of the renin–angiotensin system, calcium channel blockers, etc, have no significant effect on cognitive function. Of note, women were managed less aggressively with evidence‐based treatments for ischemic heart disease but sex differences were not examined.^101^

Noncardiac medications, such as drugs for depression, anxiety, and sleep disorders, can have significant effects on CI in both women and men, although women have smaller hippocampi and nonhippocampal brain volumes. Medications for depression, anxiety, and sleep disorders may have worse effects on CI in women.^102^

The role for endothelial dysfunction and inflammation causing accelerated atherosclerosis in collagen vascular diseases has been studied. Certain anti‐inflammatory drugs have been shown to reduce major adverse cardiovascular events.^103^ Future studies of these drugs are warranted to assess their role in CVD and CI, as well as possible sex‐related differences.

### CVD, Dementia, and Genetics

CVD and Alzheimer dementia are multifactorial diseases that also share common disease mechanisms because of an overlap in genetic^104^ and biological risk factors, which together increase the risk for CI and dementia.^105^ The APOE gene is polymorphic and encoded by 3 major alleles: APOE‐ε2, APOE‐ε3, and APOE‐ε4. The APOE‐ε4 gene is one of the most studied risk factors for CVD. Not only is the APOE protein integral to lipid metabolism and other physiological processes,^106^ but also homozygosity of the APOE e4 allele increases CAD risk by ≈40%.^104^, ^106^, ^107^, ^108^, ^109^

The APOE ε4 allele has also been associated with increased risk of developing CI and Alzheimer dementia by lowering the age of onset for both CI and dementia.^107^ Those with the APOE ε4/ε4 genotype appear to have more senile plaque density and amyloid burden than those with the APOE ε2/ε4, ε3/ε3, or ε3/ε4 genotypes.^108^ The APOE genotype also appears to differentially interact with Alzheimer dementia biomarkers in older women versus men.^109^ Lastly, there may be a stronger association of the APOE ε4 genotype with cognitive function and CI in women compared with men, suggesting a strong sex‐APOE ε4 interaction in women for risk of converting to MCI and Alzheimer dementia.^110^ Women with APOE ε3/ε4 versus men with APOE ε3/ε4 had an elevated risk, but only between the ages of 65 and 75 years.

## Summary and Conclusions

**Figure 2:**
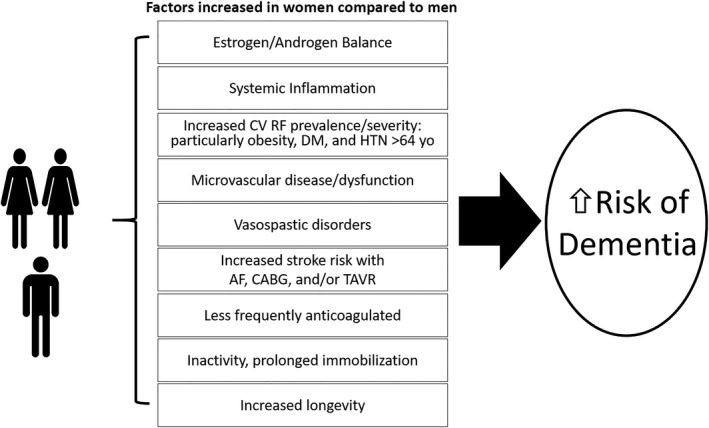
Factors that contribute to the findings that women have a higher proportion of the population with dementia. AD indicates Alzheimer disease; AF, atrial fibrillation; CABG, coronary artery bypass graft surgery; CV RF, cardiovascular risk factors; DM, diabetes mellitus; HTN, hypertension; TAVR, transcatheter aortic valve replacement.

The higher prevalence of CI and dementia in women may be explained in part by sex‐related differences in CVD risk factors, CVD and its sequelae, and CVD treatment or lack thereof. Figure 2 illustrates other factors that affect women more than men that contribute to women having a higher proportion of the population with dementia. Women have greater longevity compared with men and thus longer exposure to CVD risk factors and CVD. We summarize sex‐specific critical knowledge gaps that should be addressed in future research in Table 2. The higher prevalence of MCI in women compared with men may be ameliorated by improvements in the following: (1) stroke prevention in those with AF and hypertension; (2) appropriate treatment to prevent or reduce atherosclerosis progression; (3) better understanding of links between menopause and hypertension control; (4) work to reduce neurological complications associated with cardiac procedures; (5) avoiding menopausal hormone replacement therapy with CEE/MPA; (6) avoidance of potentially harmful medications that affect memory and cognition; and (7) understanding of the mechanistic role of APOE ε4 genotype in dementia to develop treatment targets. Analyses of sex‐specific differences in future trials are critical to improve our understanding of how CVD affects CI in women and men.

**Table 2:** Sex‐Specific Critical Knowledge Gaps

Actionable Items
Studies can be retrospectively analyzed and ongoing and future studies of cognitive function should provide sex‐specific data
Establishing whether relationships exist between coronary and cerebrovascular dysfunction to better understand links between CVD and CI/dementia
Cardiac intervention studies can be augmented to detect sex differences in detrimental effects on cognitive function
Treatment for stroke prevention such as antihypertensive medications, cholesterol‐lowering medication, and anticoagulants for AF can be assessed for their effect on sex differences in CI
Various treatments of AF can be analyzed to determine sex differences in CI
Determine whether aortic or mitral valve calcification contributes to stroke risk or a marker of increased risk and whether there are sex differences in risk of stroke
Sex differences in potential cognitive deficits with implantation of contemporary ICDs using intravenous conscious sedation and limited or no ventricular fibrillation inductions can be analyzed
Determine whether there are sex differences in interventions with therapeutic lifestyle changes to decrease cognitive impairment

AF indicates atrial fibrillation; CI, cognitive impairment; CVD, cardiovascular disease; ICD, implantable cardioverter defibrillator.

Importantly for both women and men, prevention of cognitive impairment and dementia must be viewed as a lifelong process. Therefore, to help maintain optimal brain health we endorse recommendations from national organizations such as the American Heart Association and others for promotion of cardiovascular health as a means of maintaining brain health.^111^

## Disclosures

Dr Volgman is a consultant of the American Heart Association and a speaker for Aptus Health. Dr Aggarwal has contracts between Rush University and trial sponsors to allow her to serve as a member of the Steering Committee or as site PI for the following trials: Eli Lilly: A4 Study Novartis, Amgen: Generation Study. Dr Bunch has an Institutional grant from Boehringer Ingelheim for the Cognitive Atrial Fibrillation (CAF) trial. Dr Bittner has contracts between University of Alabama and trial sponsors to allow her to serve as member of executive steering committee or as national coordinator for the following trials: Sanofi/Regeneron: ODYSSEY OUTCOMES TRIAL (Steering Committee); Astra Zeneca: STRENGTH Trial (National Coordinator); Esperion: CLEAR Trial (National Coordinator); Dalcor: Dalgene Trial (National Coordinator). She also has contracts between University of Alabama and trial sponsors to allow her to serve as a site principal investigator for the following trials: Astra Zeneca: ARTEMIS Trial, Bayer Healthcare: COMPASS Trial. She has received honoraria for attending Advisory Board meetings for Sanofi. Dr Gorelick serves on a Novartis Data Safety and Monitoring Board committee for a study of cognition in patients with heart failure utilizing the drug LCZ 696. Dr Maki is a speaker for Mylan Pharmaceutical. Dr Poppas has GE stock worth >$10 000.00. Dr Ruskin is a consultant for Acesion Pharma, Advanced Medical Education, Apple Inc., InCarda Therapeutics, Janssen, and Cala Health. He is a consultant with equity for Portola Pharmaceuticals. He is on the Scientific Advisory Board with equity options for Element Science, InfoBionic, and NewPace Medical. He is on the Scientific Advisory Board for Lantheus Medical. He is on the Steering Committee of Medtronic Pfizer. He is on the Data Monitoring Committee for Gilead Sciences. Dr Russo conducts clinical research trials for Boston Scientific, Boehringer Ingelheim, and Medtronic. She has received honoraria for consulting for Biotronik, Boston Scientific, Medtronic, and St. Jude, Zoll. She receives honorarium from Up‐to‐Date and Fellowship support from Medtronic. The remaining authors have no disclosures to report.

## References

[1] Benjamin EJ , Muntner P , Alonso A , Bittencourt MS , Callaway CW , Carson AP , Chamberlain AM , Chang AR , Cheng S , Das SR , Delling FN , Djousse L , Elkind MSV , Ferguson JF , Fornage M , Jordan LC , Khan SS , Kissela BM , Knutson KL , Kwan TW , Lackland DT , Lewis TT , Lichtman JH , Longenecker CT , Loop MS , Lutsey PL , Martin SS , Matsushita K , Moran AE , Mussolino ME , O'Flaherty M , Pandey A , Perak AM , Rosamond WD , Roth GA , Sampson UKA , Satou GM , Schroeder EB , Shah SH , Spartano NL , Stokes A , Tirschwell DL , Tsao CW , Turakhia MP , VanWagner LB , Wilkins JT , Wong SS , Virani SS . Heart disease and stroke statistics—2019 update: a report from the American Heart Association. Circulation. 2019;139:e56–e66. 30700139 10.1161/CIR.0000000000000659

[2] Alzheimer's Association 2019 Alzheimer's disease facts and figures. Alzheimers Dement. 2019;15:321–387. Available at: Alz.orghttps://www.alz.org/media/Documents/alzheimers-facts-and-figures-2019-r.pdf Accessed August 2, 2019.

[3] Schneider JA , Arvanitakis Z , Bang W , Bennett DA . Mixed brain pathologies account for most dementia cases in community‐dwelling older persons. Neurology. 2007;69:2197–2204. 17568013 10.1212/01.wnl.0000271090.28148.24

[4] Schneider JA , Arvanitakis Z , Leurgans SE , Bennett DA . The neuropathology of probable Alzheimer disease and mild cognitive impairment. Ann Neurol. 2009;66:200–208. 19743450 10.1002/ana.21706 PMC2812870

[5] Committee on family caregiving for older adults In: Schulz R, Eden J, eds. Families Caring for an Aging America. Washington, DC: National Academies Press (US); 2016: Nov. 8, 2016. Copyright 2016 by the National Academy of Sciences. All rights reserved. ISBN‐13: 978‐0‐309‐44806‐2ISBN‐10: 0‐309‐44806‐9. Available at: https://www.nap.edu/read/23606/chapter/1. 27905704

[6] McKhann GM , Knopman DS , Chertkow H , Hyman BT , Jack CR Jr , Kawas CH , Klunk WE , Koroshetz WJ , Manly JJ , Mayeux R , Mohs RC , Morris JC , Rossor MN , Scheltens P , Carrillo MC , Thies B , Weintraub S , Phelps CH . The diagnosis of dementia due to Alzheimer's disease: recommendations from the National Institute on Aging‐Alzheimer's Association workgroups on diagnostic guidelines for Alzheimer's disease. Alzheimers Dement. 2011;7:263–269. 21514250 10.1016/j.jalz.2011.03.005 PMC3312024

[7] Sperling RA , Aisen PS , Beckett LA , Bennett DA , Craft S , Fagan AM , Iwatsubo T , Jack CR Jr , Kaye J , Montine TJ , Park DC , Reiman EM , Rowe CC , Siemers E , Stern Y , Yaffe K , Carrillo MC , Thies B , Morrison‐Bogorad M , Wagster MV , Phelps CH . Toward defining the preclinical stages of Alzheimer's disease: recommendations from the National Institute on Aging‐Alzheimer's Association workgroups on diagnostic guidelines for Alzheimer's disease. Alzheimers Dement. 2011;7:280–292. 21514248 10.1016/j.jalz.2011.03.003 PMC3220946

[8] Kapasi A , DeCarli C , Schneider JA . Impact of multiple pathologies on the threshold for clinically overt dementia. Acta Neuropathol. 2017;134:171–186. 28488154 10.1007/s00401-017-1717-7 PMC5663642

[9] Jack CR Jr , Bennett DA , Blennow K , Carrillo MC , Dunn B , Haeberlein SB , Holtzman DM , Jagust W , Jessen F , Karlawish J , Liu E , Molinuevo JL , Montine T , Phelps C , Rankin KP , Rowe CC , Scheltens P , Siemers E , Snyder HM , Sperling R . NIA‐AA Research Framework: toward a biological definition of Alzheimer's disease. Alzheimers Dement. 2018;14:535–562. 29653606 10.1016/j.jalz.2018.02.018 PMC5958625

[10] Villemagne VL , Burnham S , Bourgeat P , Brown B , Ellis KA , Salvado O , Szoeke C , Macaulay SL , Martins R , Maruff P , Ames D , Rowe CC , Masters CL . Amyloid beta deposition, neurodegeneration, and cognitive decline in sporadic Alzheimer's disease: a prospective cohort study. Lancet Neurol. 2013;12:357–367. 23477989 10.1016/S1474-4422(13)70044-9

[11] Petersen RC , Lopez O , Armstrong MJ , Getchius TS , Ganguli M , Gloss D , Gronseth GS , Marson D , Pringsheim T , Day GS . Practice guideline update summary: mild cognitive impairment: report of the Guideline Development, Dissemination, and Implementation Subcommittee of the American Academy of Neurology. Neurology. 2018;90:126–135. 29282327 10.1212/WNL.0000000000004826 PMC5772157

[12] Au B , Dale‐McGrath S , Tierney MC . Sex differences in the prevalence and incidence of mild cognitive impairment: a meta‐analysis. Ageing Res Rev. 2017;35:176–199. 27771474 10.1016/j.arr.2016.09.005

[13] Chene G , Beiser A , Au R , Preis SR , Wolf PA , Dufouil C , Seshadri S . Gender and incidence of dementia in the Framingham Heart Study from mid‐adult life. Alzheimers Dement. 2015;11:310–320. 24418058 10.1016/j.jalz.2013.10.005 PMC4092061

[14] Sundermann EE , Maki PM , Rubin LH , Lipton RB , Landau S , Biegon A . Female advantage in verbal memory: evidence of sex‐specific cognitive reserve. Neurology. 2016;87:1916–1924. 27708128 10.1212/WNL.0000000000003288 PMC5100712

[15] Nebel RA , Aggarwal NT , Barnes LL , Gallagher A , Goldstein JM , Kantarci K , Mallampalli MP , Mormino EC , Scott L , Yu WH , Maki PM , Mielke MM . Understanding the impact of sex and gender in Alzheimer's disease: a call to action. Alzheimers Dement. 2018;14:1171–1183. 29907423 10.1016/j.jalz.2018.04.008 PMC6400070

[16] Pool LR , Weuve J , Wilson RS , Bultmann U , Evans DA , Mendes de Leon CF . Occupational cognitive requirements and late‐life cognitive aging. Neurology. 2016;86:1386–1392. 26984944 10.1212/WNL.0000000000002569 PMC4831043

[17] Fisher GG , Stachowski A , Infurna FJ , Faul JD , Grosch J , Tetrick LE . Mental work demands, retirement, and longitudinal trajectories of cognitive functioning. J Occup Health Psychol. 2014;19:231–242. 24635733 10.1037/a0035724 PMC4663987

[18] Kasper JD , Freedman VA , Spillman BC , Wolff JL . The disproportionate impact of dementia on family and unpaid caregiving to older adults. Health Aff. 2015;34:1642–1649. 10.1377/hlthaff.2015.0536 PMC4635557 26438739

[19] Elias MF , Goodell AL , Dore GA . Hypertension and cognitive functioning: a perspective in historical context. Hypertension. 2012;60:260–268. 22753214 10.1161/HYPERTENSIONAHA.111.186429

[20] Iadecola C , Yaffe K , Biller J , Bratzke LC , Faraci FM , Gorelick PB , Gulati M , Kamel H , Knopman DS , Launer LJ , Saczynski JS , Seshadri S , Zeki Al Hazzouri A . Impact of hypertension on cognitive function: a scientific statement from the American Heart Association. Hypertension. 2016;68:e67–e94. 27977393 10.1161/HYP.0000000000000053 PMC5361411

[21] Walker KA , Power MC , Gottesman RF . Defining the relationship between hypertension, cognitive decline, and dementia: a review. Curr Hypertens Rep. 2017;19:24. 28299725 10.1007/s11906-017-0724-3 PMC6164165

[22] Perrotta M , Lembo G , Carnevale D . Hypertension and dementia: epidemiological and experimental evidence revealing a detrimental relationship. Int J Mol Sci. 2016;17:347. 27005613 10.3390/ijms17030347 PMC4813208

[23] Waldstein SR , Wendell CR . Neurocognitive function and cardiovascular disease. J Alzheimers Dis. 2010;20:833–842. 20413878 10.3233/JAD-2010-091591

[24] Waldstein SR . Hypertension and neuropsychological function: a lifespan perspective. Exp Aging Res. 1995;21:321–352. 8595801 10.1080/03610739508253989

[25] Lande MB , Kupferman JC , Adams HR . Neurocognitive alterations in hypertensive children and adolescents. J Clin Hypertens (Greenwich). 2012;14:353–359. 22672088 10.1111/j.1751-7176.2012.00661.x PMC3375877

[26] Rovio SP , Pahkala K , Nevalainen J , Juonala M , Salo P , Kähönen M , Hutri‐Kähönen N , Lehtimäki T , Jokinen E , Laitinen T . Cardiovascular risk factors from childhood and midlife cognitive performance: the Young Finns Study. J Am Coll Cardiol. 2017;69:2279–2289. 28473132 10.1016/j.jacc.2017.02.060

[27] Waldstein SR , Manuck SB , Ryan CM , Muldoon MF . Neuropsychological correlates of hypertension: review and methodologic considerations. Psychol Bull. 1991;110:451. 1758919 10.1037/0033-2909.110.3.451

[28] Rouch L , Cestac P , Hanon O , Cool C , Helmer C , Bouhanick B , Chamontin B , Dartigues JF , Vellas B , Andrieu S . Antihypertensive drugs, prevention of cognitive decline and dementia: a systematic review of observational studies, randomized controlled trials and meta‐analyses, with discussion of potential mechanisms. CNS Drugs. 2015;29:113–130. 25700645 10.1007/s40263-015-0230-6

[29] Kjeldsen SE , Narkiewicz K , Burnier M , Oparil S . Intensive blood pressure lowering prevents mild cognitive impairment and possible dementia and slows development of white matter lesions in brain: the SPRINT Memory and Cognition IN Decreased Hypertension (SPRINT MIND) study. Blood Press. 2018;27:247. 30175661 10.1080/08037051.2018.1507621

[30] Williamson JD , Launer LJ , Bryan RN , Coker LH , Lazar RM , Gerstein HC , Murray AM , Sullivan MD , Horowitz KR , Ding J , Marcovina S , Lovato L , Lovato J , Margolis KL , Davatzikos C , Barzilay J , Ginsberg HN , Linz PE , Miller ME ; Action to Control Cardiovascular Risk in Diabetes Memory in Diabetes Investigators . Cognitive function and brain structure in persons with type 2 diabetes mellitus after intensive lowering of blood pressure and lipid levels: a randomized clinical trial. JAMA Intern Med. 2014;174:324–333. 24493100 10.1001/jamainternmed.2013.13656 PMC4423790

[31] Kritz‐Silverstein D , Laughlin GA , McEvoy LK , Barrett‐Connor E . Sex and age differences in the association of blood pressure and hypertension with cognitive function in the elderly: the Rancho Bernardo Study. J Prev Alzheimers Dis. 2017;4:165–173. 29182707 10.14283/jpad.2017.6 PMC5706556

[32] Gilsanz P , Mayeda ER , Glymour MM , Quesenberry CP , Mungas DM , DeCarli C , Dean A , Whitmer RA . Female sex, early‐onset hypertension, and risk of dementia. Neurology. 2017;89:1886–1893. 28978656 10.1212/WNL.0000000000004602 PMC5664296

[33] Zilberman JM , Cerezo GH , Del Sueldo M , Fernandez‐Perez C , Martell‐Claros N , Vicario A . Association between hypertension, menopause, and cognition in women. J Clin Hypertens (Greenwich). 2015;17:970–976. 26252810 10.1111/jch.12643 PMC8031519

[34] Stefanidis KB , Askew CD , Greaves K , Summers MJ . The effect of non‐stroke cardiovascular disease states on risk for cognitive decline and dementia: a systematic and meta‐analytic review. Neuropsychol Rev. 2018;28:1–15. 28856507 10.1007/s11065-017-9359-z

[35] Gorelick PB , Wong KS , Bae HJ , Pandey DK . Large artery intracranial occlusive disease: a large worldwide burden but a relatively neglected frontier. Stroke. 2008;39:2396–2399. 18535283 10.1161/STROKEAHA.107.505776

[36] Gorelick PB . Distribution of atherosclerotic cerebrovascular lesions. Effects of age, race, and sex. Stroke. 1993;24:I16–I19; discussion I20–1. 8249013

[37] Joakimsen O , Bonaa KH , Stensland‐Bugge E , Jacobsen BK . Population‐based study of age at menopause and ultrasound assessed carotid atherosclerosis: the Tromso Study. J Clin Epidemiol. 2000;53:525–530. 10812326 10.1016/s0895-4356(99)00197-3

[38] Pu Y , Liu L , Wang Y , Zou X , Pan Y , Soo Y , Leung T , Zhao X , Wong KS , Wang Y . Geographic and sex difference in the distribution of intracranial atherosclerosis in China. Stroke. 2013;44:2109–2114. 23760212 10.1161/STROKEAHA.113.001522

[39] Kasner SE , Chimowitz MI , Lynn MJ , Howlett‐Smith H , Stern BJ , Hertzberg VS , Frankel MR , Levine SR , Chaturvedi S , Benesch CG , Sila CA , Jovin TG , Romano JG , Cloft HJ . Predictors of ischemic stroke in the territory of a symptomatic intracranial arterial stenosis. Circulation. 2006;113:555–563. 16432056 10.1161/CIRCULATIONAHA.105.578229

[40] Knopman D , Boland LL , Mosley T , Howard G , Liao D , Szklo M , McGovern P , Folsom AR . Cardiovascular risk factors and cognitive decline in middle‐aged adults. Neurology. 2001;56:42–48. 11148234 10.1212/wnl.56.1.42

[41] Luchsinger JA , Reitz C , Honig LS , Tang MX , Shea S , Mayeux R . Aggregation of vascular risk factors and risk of incident Alzheimer disease. Neurology. 2005;65:545–551. 16116114 10.1212/01.wnl.0000172914.08967.dc PMC1619350

[42] Gorelick PB , Scuteri A , Black SE , Decarli C , Greenberg SM , Iadecola C , Launer LJ , Laurent S , Lopez OL , Nyenhuis D , Petersen RC , Schneider JA , Tzourio C , Arnett DK , Bennett DA , Chui HC , Higashida RT , Lindquist R , Nilsson PM , Roman GC , Sellke FW , Seshadri S . Vascular contributions to cognitive impairment and dementia: a statement for healthcare professionals from the American Heart Association/American Stroke Association. Stroke. 2011;42:2672–2713. 21778438 10.1161/STR.0b013e3182299496 PMC3778669

[43] Roher AE , Esh C , Kokjohn TA , Kalback W , Luehrs DC , Seward JD , Sue LI , Beach TG . Circle of Willis atherosclerosis is a risk factor for sporadic Alzheimer's disease. Arterioscler Thromb Vasc Biol. 2003;23:2055–2062. 14512367 10.1161/01.ATV.0000095973.42032.44

[44] Kalaria RN , Akinyemi R , Ihara M . Does vascular pathology contribute to Alzheimer changes? J Neurol Sci. 2012;322:141–147. 22884479 10.1016/j.jns.2012.07.032

[45] Bos D , van der Rijk MJ , Geeraedts TE , Hofman A , Krestin GP , Witteman JC , van der Lugt A , Ikram MA , Vernooij MW . Intracranial carotid artery atherosclerosis: prevalence and risk factors in the general population. Stroke. 2012;43:1878–1884. 22569939 10.1161/STROKEAHA.111.648667

[46] Taqueti VR . Sex differences in the coronary system. Adv Exp Med Biol. 2018;1065:257–278. 30051390 10.1007/978-3-319-77932-4_17 PMC6467060

[47] Sweeney MD , Montagne A , Sagare AP , Nation DA , Schneider LS , Chui HC , Harrington MG , Pa J , Law M , Wang DJJ , Jacobs RE , Doubal FN , Ramirez J , Black SE , Nedergaard M , Benveniste H , Dichgans M , Iadecola C , Love S , Bath PM , Markus HS , Salman RA , Allan SM , Quinn TJ , Kalaria RN , Werring DJ , Carare RO , Touyz RM , Williams SCR , Moskowitz MA , Katusic ZS , Lutz SE , Lazarov O , Minshall RD , Rehman J , Davis TP , Wellington CL , Gonzalez HM , Yuan C , Lockhart SN , Hughes TM , Chen CLH , Sachdev P , O'Brien JT , Skoog I , Pantoni L , Gustafson DR , Biessels GJ , Wallin A , Smith EE , Mok V , Wong A , Passmore P , Barkof F , Muller M , Breteler MMB , Roman GC , Hamel E , Seshadri S , Gottesman RF , van Buchem MA , Arvanitakis Z , Schneider JA , Drewes LR , Hachinski V , Finch CE , Toga AW , Wardlaw JM , Zlokovic BV . Vascular dysfunction—the disregarded partner of Alzheimer's disease. Alzheimers Dement. 2019;15:158–167. 30642436 10.1016/j.jalz.2018.07.222 PMC6338083

[48] Vidal JS , Sigurdsson S , Jonsdottir MK , Eiriksdottir G , Thorgeirsson G , Kjartansson O , Garcia ME , van Buchem MA , Harris TB , Gudnason V , Launer LJ . Coronary artery calcium, brain function and structure: the AGES‐Reykjavik Study. Stroke. 2010;41:891–897. 20360538 10.1161/STROKEAHA.110.579581 PMC3298743

[49] Bos D , Vernooij MW , de Bruijn RF , Koudstaal PJ , Hofman A , Franco OH , van der Lugt A , Ikram MA . Atherosclerotic calcification is related to a higher risk of dementia and cognitive decline. Alzheimers Dement. 2015;11:639–647.e1. 25150731 10.1016/j.jalz.2014.05.1758

[50] Schmidt KMT , Nan J , Scantlebury DC , Aggarwal NR . Stable ischemic heart disease in women. Curr Treat Options Cardiovasc Med. 2018;20:72. 30084006 10.1007/s11936-018-0665-4

[51] Taqueti VR , Di Carli MF . Coronary microvascular disease pathogenic mechanisms and therapeutic options: JACC state‐of‐the‐art review. J Am Coll Cardiol. 2018;72:2625–2641. 30466521 10.1016/j.jacc.2018.09.042 PMC6296779

[52] Roberts RO , Knopman DS , Geda YE , Cha RH , Roger VL , Petersen RC . Coronary heart disease is associated with non‐amnestic mild cognitive impairment. Neurobiol Aging. 2010;31:1894–1902. 19091445 10.1016/j.neurobiolaging.2008.10.018 PMC2888961

[53] Ahto M , Isoaho R , Puolijoki H , Laippala P , Sulkava R , Kivela SL . Cognitive impairment among elderly coronary heart disease patients. Gerontology. 1999;45:87–95. 9933731 10.1159/000022069

[54] Gharacholou SM , Reid KJ , Arnold SV , Spertus J , Rich MW , Pellikka PA , Singh M , Holsinger T , Krumholz HM , Peterson ED , Alexander KP . Cognitive impairment and outcomes in older adult survivors of acute myocardial infarction: findings from the translational research investigating underlying disparities in acute myocardial infarction patients’ health status registry. Am Heart J. 2011;162:860–869.e1. 22093202 10.1016/j.ahj.2011.08.005 PMC3410733

[55] Golive A , May HT , Bair TL , Jacobs V , Crandall BG , Cutler MJ , Day JD , Mallender C , Osborn JS , Weiss JP , Bunch TJ . The impact of gender on atrial fibrillation incidence and progression to dementia. Am J Cardiol. 2018;122:1489–1495. 30195396 10.1016/j.amjcard.2018.07.031

[56] Kwok CS , Loke YK , Hale R , Potter JF , Myint PK . Atrial fibrillation and incidence of dementia: a systematic review and meta‐analysis. Neurology. 2011;76:914–922. 21383328 10.1212/WNL.0b013e31820f2e38

[57] Kalantarian S , Stern TA , Mansour M , Ruskin JN . Cognitive impairment associated with atrial fibrillation: a meta‐analysis. Ann Intern Med. 2013;158:338–346. 23460057 10.7326/0003-4819-158-5-201303050-00007 PMC4465526

[58] Reeves MJ , Bushnell CD , Howard G , Gargano JW , Duncan PW , Lynch G , Khatiwoda A , Lisabeth L . Sex differences in stroke: epidemiology, clinical presentation, medical care, and outcomes. Lancet Neurol. 2008;7:915–926. 18722812 10.1016/S1474-4422(08)70193-5 PMC2665267

[59] Gargano JW , Reeves MJ ; Paul Coverdell National Acute Stroke Registry Michigan Prototype Investigators . Sex differences in stroke recovery and stroke‐specific quality of life: results from a statewide stroke registry. Stroke. 2007;38:2541–2548. 17673706 10.1161/STROKEAHA.107.485482

[60] Jacobs V , Woller SC , Stevens S , May HT , Bair TL , Anderson JL , Crandall BG , Day JD , Johanning K , Long Y . Time outside of therapeutic range in atrial fibrillation patients is associated with long‐term risk of dementia. Heart Rhythm. 2014;11:2206–2213. 25111326 10.1016/j.hrthm.2014.08.013

[61] Jacobs V , May HT , Bair TL , Crandall BG , Cutler MJ , Day JD , Mallender C , Osborn JS , Stevens SM , Weiss JP , Woller SC , Bunch TJ . Long‐term population‐based cerebral ischemic event and cognitive outcomes of direct oral anticoagulants compared with warfarin among long‐term anticoagulated patients for atrial fibrillation. Am J Cardiol. 2016;118:210–214. 27236255 10.1016/j.amjcard.2016.04.039

[62] Thompson LE , Maddox TM , Lei L , Grunwald GK , Bradley SM , Peterson PN , Masoudi FA , Turchin A , Song Y , Doros G , Davis MB , Daugherty SL . Sex differences in the use of oral anticoagulants for atrial fibrillation: a report from the National Cardiovascular Data Registry (NCDR^®^) PINNACLE Registry. J Am Heart Assoc. 2017;6:e005801 DOI: 10.1161/JAHA.117.005801. 28724655 PMC5586299

[63] National Institute of Health . Guidelines on the inclusion of women and minorities as subjects in clinical research. 1994;59:14504–14513.

[64] Graves KG , May HT , Jacobs V , Bair TL , Stevens SM , Woller SC , Crandall BG , Cutler MJ , Day JD , Mallender C , Osborn JS , Peter Weiss J , Jared Bunch T . Atrial fibrillation incrementally increases dementia risk across all CHADS2 and CHA2DS2VASc strata in patients receiving long‐term warfarin. Am Heart J. 2017;188:93–98. 28577686 10.1016/j.ahj.2017.02.026

[65] Kalantarian S , Ay H , Gollub RL , Lee H , Retzepi K , Mansour M , Ruskin JN . Association between atrial fibrillation and silent cerebral infarctions: a systematic review and meta‐analysis. Ann Intern Med. 2014;161:650–658. 25364886 10.7326/M14-0538 PMC5578742

[66] Gaita F , Corsinovi L , Anselmino M , Raimondo C , Pianelli M , Toso E , Bergamasco L , Boffano C , Valentini MC , Cesarani F , Scaglione M . Prevalence of silent cerebral ischemia in paroxysmal and persistent atrial fibrillation and correlation with cognitive function. J Am Coll Cardiol. 2013;62:1990–1997. 23850917 10.1016/j.jacc.2013.05.074

[67] Friberg L , Rosenqvist M . Less dementia with oral anticoagulation in atrial fibrillation. Eur Heart J. 2018;39:453–460. 29077849 10.1093/eurheartj/ehx579

[68] January CT , Wann LS , Calkins H , Chen LY , Cigarroa JE , Cleveland JC Jr , Ellinor PT , Ezekowitz MD , Field ME , Furie KL , Heidenreich PA , Murray KT , Shea JB , Tracy CM , Yancy CW . 2019 AHA/ACC/HRS focused update of the 2014 AHA/ACC/HRS guideline for the management of patients with atrial fibrillation. Circulation. 2019;140:e125–e151. 30686041 10.1161/CIR.0000000000000665

[69] Leng Y , McEvoy CT , Allen IE , Yaffe K . Association of sleep‐disordered breathing with cognitive function and risk of cognitive impairment: a systematic review and meta‐analysis. JAMA Neurol. 2017;74:1237–1245. 28846764 10.1001/jamaneurol.2017.2180 PMC5710301

[70] Chiu HY , Lai FC , Chen PY , Tsai PS . Differences between men and women aged 65 and older in the relationship between self‐reported sleep and cognitive impairment: a nationwide survey in Taiwan. J Am Geriatr Soc. 2016;64:2051–2058. 27627762 10.1111/jgs.14316

[71] Vogels RL , Scheltens P , Schroeder‐Tanka JM , Weinstein HC . Cognitive impairment in heart failure: a systematic review of the literature. Eur J Heart Fail. 2007;9:440–449. 17174152 10.1016/j.ejheart.2006.11.001

[72] Bennett SJ , Sauvé MJ . Cognitive deficits in patients with heart failure: a review of the literature. J Cardiovasc Nurs. 2003;18:219–242. 12837012 10.1097/00005082-200307000-00007

[73] Gure TR , Blaum CS , Giordani B , Koelling TM , Galecki A , Pressler SJ , Hummel SL , Langa KM . Prevalence of cognitive impairment in older adults with heart failure. J Am Geriatr Soc. 2012;60:1724–1729. 22882000 10.1111/j.1532-5415.2012.04097.x PMC3445700

[74] Tibrewala A , Yancy CW . Heart failure with preserved ejection fraction in women. Heart Fail Clin. 2019;15:9–18. 30449384 10.1016/j.hfc.2018.08.002

[75] Cogswell RJ , Norby FL , Gottesman RF , Chen LY , Solomon S , Shah A , Alonso A . High prevalence of subclinical cerebral infarction in patients with heart failure with preserved ejection fraction. Eur J Heart Fail. 2017;19:1303–1309. 28738140 10.1002/ejhf.812 PMC5933437

[76] Dolansky MA , Hawkins MA , Schaefer JT , Sattar A , Gunstad J , Redle JD , Josephson R , Moore SM , Hughes JW . Association between poorer cognitive function and reduced objectively monitored medication adherence in patients with heart failure. Circ Heart Fail. 2016;9:e002475. 27895069 10.1161/CIRCHEARTFAILURE.116.002475 PMC5131517

[77] Gress DR . The problem with asymptomatic cerebral embolic complications in vascular procedures: what if they are not asymptomatic? J Am Coll Cardiol. 2012;60:1614–1616. 22999732 10.1016/j.jacc.2012.06.037

[78] Kodali S , Williams MR , Doshi D , Hahn RT , Humphries KH , Nkomo VT , Cohen DJ , Douglas PS , Mack M , Xu K , Svensson L , Thourani VH , Tuzcu EM , Weissman NJ , Leon M , Kirtane AJ . Sex‐specific differences at presentation and outcomes among patients undergoing transcatheter aortic valve replacement: a cohort study. Ann Intern Med. 2016;164:377–384. 26903039 10.7326/M15-0121

[79] Chaker Z , Badhwar V , Alqahtani F , Aljohani S , Zack CJ , Holmes DR , Rihal CS , Alkhouli M . Sex differences in the utilization and outcomes of surgical aortic valve replacement for severe aortic stenosis. J Am Heart Assoc. 2017;6:e006370 DOI: 10.1161/JAHA.117.006370. 28935681 PMC5634288

[80] Goto T , Baba T , Ito A , Maekawa K , Koshiji T . Gender differences in stroke risk among the elderly after coronary artery surgery. Anesth Analg. 2007;104:1016–1022, tables of contents. 17456646 10.1213/01.ane.0000263279.07361.1f

[81] Auffret V , Campelo‐Parada F , Regueiro A , Del Trigo M , Chiche O , Chamandi C , Allende R , Cordoba‐Soriano JG , Paradis JM , De Larochelliere R , Doyle D , Dumont E , Mohammadi S , Cote M , Marrero A , Puri R , Rodes‐Cabau J . Serial changes in cognitive function following transcatheter aortic valve replacement. J Am Coll Cardiol. 2016;68:2129–2141. 27692728 10.1016/j.jacc.2016.08.046

[82] Itchhaporia D . Transcatheter aortic valve replacement in women. Clin Cardiol. 2018;41:228–231. 29485678 10.1002/clc.22912 PMC6489724

[83] Singer I , van der Laken J , Edmonds HL Jr , Slater AD , Austin E , Shields CB , Kupersmith J . Is defibrillation testing safe? Pacing Clin Electrophysiol. 1991;14:1899–1904. 1721196 10.1111/j.1540-8159.1991.tb02787.x

[84] Vriens EM , Bakker PF , Vries JW , Wieneke GH , Van Huffelen AC . The impact of repeated short episodes of circulatory arrest on cerebral function. Reassuring electroencephalographic (EEG) findings during defibrillation threshold testing at defibrillator implantation. Electroencephalogr Clin Neurophysiol. 1996;98:236–242. 8641146 10.1016/0013-4694(95)00248-0

[85] Halas K , Krzyzanowski K , Krzyzanowska E , Smurzynski P , Ryczek R , Michalkiewicz D , Orski Z , Makowski K , Wierzbowski R , Gielerak G . Cognitive impairment after appropriate implantable cardioverter‐defibrillator therapy for ventricular fibrillation. Kardiol Pol. 2014;72:134–139. 23990235 10.5603/KP.a2013.0215

[86] Benedini G , Marchini A , Curnis A , Bianchetti F , Gardini A , Pinetti P , Zanelli E . Implantable defibrillation and thromboembolic events. Pacing Clin Electrophysiol. 1995;18:199–202. 7724399 10.1111/j.1540-8159.1995.tb02504.x

[87] Edwards FH , Carey JS , Grover FL , Bero JW , Hartz RS . Impact of gender on coronary bypass operative mortality. Ann Thorac Surg. 1998;66:125–131. 9692451 10.1016/s0003-4975(98)00358-0

[88] Khan SS , Nessim S , Gray R , Czer LS , Chaux A , Matloff J . Increased mortality of women in coronary artery bypass surgery: evidence for referral bias. Ann Intern Med. 1990;112:561–567. 2327676 10.7326/0003-4819-112-8-561

[89] Pina IL , Zheng Q , She L , Szwed H , Lang IM , Farsky PS , Castelvecchio S , Biernat J , Paraforos A , Kosevic D , Favaloro LE , Nicolau JC , Varadarajan P , Velazquez EJ , Pai RG , Cyrille N , Lee KL , Desvigne‐Nickens P . Sex difference in patients with ischemic heart failure undergoing surgical revascularization: results from the STICH Trial (Surgical Treatment for Ischemic Heart Failure). Circulation. 2018;137:771–780. 29459462 10.1161/CIRCULATIONAHA.117.030526 PMC5896331

[90] Hogue CW Jr , Barzilai B , Pieper KS , Coombs LP , DeLong ER , Kouchoukos NT , Davila‐Roman VG . Sex differences in neurological outcomes and mortality after cardiac surgery: a society of thoracic surgery national database report. Circulation. 2001;103:2133–2137. 11331252 10.1161/01.cir.103.17.2133

[91] Newman MF , Kirchner JL , Phillips‐Bute B , Gaver V , Grocott H , Jones RH , Mark DB , Reves JG , Blumenthal JA . Longitudinal assessment of neurocognitive function after coronary‐artery bypass surgery. N Engl J Med. 2001;344:395–402. 11172175 10.1056/NEJM200102083440601

[92] Kennedy ED , Choy KC , Alston RP , Chen S , Farhan‐Alanie MM , Anderson J , Ang YL , Moore DE , MacKenzie SA , Sykes RA . Cognitive outcome after on‐and off‐pump coronary artery bypass grafting surgery: a systematic review and meta‐analysis. J Cardiothorac Vasc Anesth. 2013;27:253–265. 23507014 10.1053/j.jvca.2012.11.008

[93] Benvenuti SM , Patron E , Zanatta P , Polesel E , Bonfà C , Palomba D . Change in behavioral functional capacity is associated with preexisting cognitive function rather than with cognitive decline in patients 1 year after cardiac surgery. Gen Hosp Psychiatry. 2013;35:117–121. 23351525 10.1016/j.genhosppsych.2012.12.008

[94] Hogue CW , Lillie R , Hershey T , Birge S , Nassief AM , Thomas B , Freedland KE . Gender influence on cognitive function after cardiac operation. Ann Thorac Surg. 2003;76:1119–1125. 14529997 10.1016/s0003-4975(03)00817-8

[95] Elharram M , Dayan N , Kaur A , Landry T , Pilote L . Long‐term cognitive impairment after preeclampsia: a systematic review and meta‐analysis. Obstet Gynecol. 2018;132:355–364. 29995746 10.1097/AOG.0000000000002686

[96] Shumaker S , Legault C , Kuller L , Rapp S , Thal L , Lane D , Fillit H , Stefanick M , Hendrix S , Lewis C , Masaki K , Coker L ; Women's Health Initiative Memory Study . Conjugated equine estrogens and incidence of probable dementia and mild cognitive impairment in postmenopausal women: Women's Health Initiative Memory Study. JAMA. 2004;291:2947–2958. 15213206 10.1001/jama.291.24.2947

[97] Coker LH , Espeland MA , Hogan PE , Resnick SM , Bryan RN , Robinson JG , Goveas JS , Davatzikos C , Kuller LH , Williamson JD , Bushnell CD , Shumaker SA ; WHIMS‐MRI Study Group . Change in brain and lesion volumes after CEE therapies: the WHIMS‐MRI studies. Neurology. 2014;82:427–434. 24384646 10.1212/WNL.0000000000000079 PMC3917682

[98] Rossouw JE , Anderson GL , Prentice RL , LaCroix AZ , Kooperberg C , Stefanick ML , Jackson RD , Beresford SA , Howard BV , Johnson KC , Kotchen JM , Ockene J . Risks and benefits of estrogen plus progestin in healthy postmenopausal women: principal results from the Women's Health Initiative randomized controlled trial. JAMA. 2002;288:321–333. 12117397 10.1001/jama.288.3.321

[99] Manson JE , Aragaki AK , Rossouw JE , Anderson GL , Prentice RL , LaCroix AZ , Chlebowski RT , Howard BV , Thomson CA , Margolis KL , Lewis CE , Stefanick ML , Jackson RD , Johnson KC , Martin LW , Shumaker SA , Espeland MA , Wactawski‐Wende J ; WHI Investigators . Menopausal hormone therapy and long‐term all‐cause and cause‐specific mortality: the Women's Health Initiative randomized trials. JAMA. 2017;318:927–938. 28898378 10.1001/jama.2017.11217 PMC5728370

[100] Espeland MA , Brinton RD , Hugenschmidt C , Manson JE , Craft S , Yaffe K , Weitlauf J , Vaughan L , Johnson KC , Padula CB , Jackson RD , Resnick SM ; WHIMS Study Group . Impact of type 2 diabetes and postmenopausal hormone therapy on incidence of cognitive impairment in older women. Diabetes Care. 2015;38:2316–2324. 26486190 10.2337/dc15-1385 PMC4657616

[101] Aggarwal NR , Patel HN , Mehta LS , Sanghani RM , Lundberg GP , Lewis SJ , Mendelson MA , Wood MJ , Volgman AS , Mieres JH . Sex differences in ischemic heart disease: advances, obstacles, and next steps. Circ Cardiovasc Qual Outcomes. 2018;11:e004437. 29449443 10.1161/CIRCOUTCOMES.117.004437

[102] Daulatzai MA . Neurotoxic saboteurs: straws that break the hippo's (hippocampus) back drive cognitive impairment and Alzheimer's disease. Neurotox Res. 2013;24:407–459. 23820984 10.1007/s12640-013-9407-2

[103] Aday AW , Ridker PM . Targeting residual inflammatory risk: a shifting paradigm for atherosclerotic disease. Front Cardiovasc Med. 2019;6:16. 30873416 10.3389/fcvm.2019.00016 PMC6403155

[104] Kotze MJ , Van Rensburg SJ . Pathology supported genetic testing and treatment of cardiovascular disease in middle age for prevention of Alzheimer's disease. Metab Brain Dis. 2012;27:255–266. 22552896 10.1007/s11011-012-9296-8 PMC3429783

[105] Whitmer RA , Gunderson EP , Barrett‐Connor E , Quesenberry CP , Yaffe K . Obesity in middle age and future risk of dementia: a 27 year longitudinal population based study. BMJ. 2005;330:1360. 15863436 10.1136/bmj.38446.466238.E0 PMC558283

[106] Davignon J . Apolipoprotein E polymorphism and atherosclerosis. New horizons in coronary heart disease Schwartz , Colin John and Born , Gustav VR (Eds) Volume 5:1–5. London, England: Current Science, 1993.

[107] Saunders AM , Strittmatter WJ , Schmechel D , George‐Hyslop PS , Pericak‐Vance M , Joo S , Rosi B , Gusella J , Crapper‐MacLachlan D , Alberts M . Association of apolipoprotein E allele ε4 with late‐onset familial and sporadic Alzheimer's disease. Neurology. 1993;43:1467–1472. 8350998 10.1212/wnl.43.8.1467

[108] Yip AG , McKee AC , Green RC , Wells J , Young H , Cupples LA , Farrer LA . APOE, vascular pathology, and the AD brain. Neurology. 2005;65:259–265. 16043796 10.1212/01.wnl.0000168863.49053.4d

[109] Koran MEI , Wagener M , Hohman TJ ; Alzheimer's Neuroimaging I . Sex differences in the association between AD biomarkers and cognitive decline. Brain Imaging Behav. 2017;11:205–213. 26843008 10.1007/s11682-016-9523-8 PMC4972701

[110] Altmann A , Tian L , Henderson VW , Greicius MD ; Alzheimer's Disease Neuroimaging Initiative I . Sex modifies the APOE‐related risk of developing Alzheimer disease. Ann Neurol. 2014;75:563–573. 24623176 10.1002/ana.24135 PMC4117990

[111] Gorelick PB , Furie KL , Iadecola C , Smith EE , Waddy SP , Lloyd‐Jones DM , Bae HJ , Bauman MA , Dichgans M , Duncan PW , Girgus M , Howard VJ , Lazar RM , Seshadri S , Testai FD , van Gaal S , Yaffe K , Wasiak H , Zerna C . Defining optimal brain health in adults: a presidential advisory from the American Heart Association/American Stroke Association. Stroke. 2017;48:e284–e303. 28883125 10.1161/STR.0000000000000148 PMC5654545

